# IgG Glycosylation Analysis in Patients with Ring14 Syndrome Unveils Novel Pathomechanisms and New Therapy Perspectives

**DOI:** 10.3390/biom16060760

**Published:** 2026-05-22

**Authors:** Angela Messina, Angelo Palmigiano, Donata Agata Romeo, Luisa Sturiale, Enrico Parano, Marco Crimi, Annunziata Carrese Cirillo, Alessandro Vaisfeld, Rita Barone, Domenico Garozzo

**Affiliations:** 1 CNR—Institute for Polymers, Composites and Biomaterials (IPCB), 95126 Catania, Italy; angela.messina@cnr.it (A.M.); angelo.palmigiano@cnr.it (A.P.); donataagata.romeo@cnr.it (D.A.R.); luisella.sturiale@cnr.it (L.S.); 2 CNR—Institute for Biomedical Research and Innovation (IRIB), 95126 Catania, Italy; enrico.parano@cnr.it; 3 Ring14 Italy, Via Flavio Gioia 5, 42124 Reggio Emilia, Italy; crimi.marco@ring14.org; 4 Kaleidos SCS, Scientific Office, Via Andrea Moretti, 24121 Bergamo, Italy; 5 Azienda Unità Sanitaria Locale di Reggio Emilia, 42122 Reggio Emilia, Italy; annunziata.carresecirillo@ausl.re.it; 6 Department of Medical and Surgical Sciences (DIMEC), Alma Mater Studiorum-University of Bologna, 40126 Bologna, Italy; alessandro.vaisfeld2@unibo.it; 7 Medical Genetics Unit, IRCCS Azienda Ospedaliero-Universitaria di Bologna, 40138 Bologna, Italy; 8 Child Neurology and Psychiatry Section, Department of Clinical and Experimental Medicine, University of Catania, 95124 Catania, Italy; rbarone@unict.it

**Keywords:** Ring14, N-glycans, IgG, CDG, IVIG, Epilepsy

## Abstract

Ring chromosome 14 (RC14) syndrome is an ultra-rare disorder characterized by drug-resistant epilepsy, intellectual disabilities, autism, and recurrent infections, suggesting a possible underlying immune dysregulation. We analyzed immunoglobulin G (IgG) N-glycosylation profiles in six RC14 patients and compared them with age-matched healthy controls using ultra-high-performance liquid chromatography (UHPLC) coupled with fluorescence detection (FLR) and high-resolution electrospray ionization mass spectrometry (ESI-MS). Patients showed decreased galactosylation and sialylation, resembling pro-inflammatory patterns observed in autoimmune diseases. These alterations were not observed in total serum glycoproteins, indicating a selective effect on IgG. One patient treated with intravenous immunoglobulin (IVIG) showed clinical improvement, which led us to investigate causality.

## 1. Introduction

Ring chromosome 14 (RC14) syndrome is an ultra-rare chromosomal disorder that results from a structural rearrangement of chromosome 14, ultimately giving rise to a ring configuration that compromises genomic integrity. The formation of the ring chromosome induces mitotic instability and leads to variable terminal deletions, thereby generating a heterogeneous molecular background that contributes to the broad clinical variability observed among affected individuals.

The syndrome was originally described in 1971 by Gilgenkrantz and colleagues [1], whose report established the foundation for subsequent clinical and cytogenetic investigations. Despite this early recognition, RC14 syndrome has remained insufficiently characterized, largely because its extreme rarity and the complexity of its genomic architecture limit the availability of systematic cohort studies. Consequently, true prevalence and incidence remain undetermined, and fewer than 100 cases have been documented worldwide [2]. Reported patients exhibit considerable variability in the size of the terminally deleted region, which may range from sub-megabase losses to several megabases, further complicating genotype–phenotype correlations. Clinically, RC14 syndrome encompasses a recognizable constellation of features, including distinctive craniofacial characteristics, developmental delay progressing to severe intellectual disability, postnatal microcephaly, retinal abnormalities, and highly drug-resistant epilepsy [3,4]. These manifestations reflect the combined effects of chromosomal instability, haploinsufficiency of genes located on 14q, and potential epigenetic dysregulation associated with ring formation.

A prominent and clinically relevant aspect of RC14 syndrome is the marked susceptibility to infections, particularly severe and recurrent respiratory infections that frequently necessitate hospitalization and represent a major cause of mortality. Although factors such as chronic hypoxia, feeding difficulties, and aspiration risk may contribute to this vulnerability, emerging evidence indicates that immune dysregulation plays a substantive role. Recent clinical cohorts have reported alterations in immune cell subsets and impaired immune responses [2,5], suggesting that the immunological phenotype may be an intrinsic component of the disorder rather than a secondary consequence of neurological impairment.

Furthermore, fever episodes may trigger rapid clinical deterioration, especially in the presence of comorbidities such as epilepsy or malnutrition, supporting an improper immunological response in RC14 patients. As immunoglobulin G (IgG) levels remain consistent, functional, rather than quantitative IgG abnormalities, may be implicated, such as aberrant glycosylation, since it can profoundly influence IgG function [6,7]. Protein N-glycosylation is the most common glycosylation process in humans, with about 80–90% of glycoproteins being N-glycosylated. In the N-glycosylation pathway, a glycan is linked to the nitrogen of an asparagine (Asn) residue, usually within an “Asn-X-Ser/Thr” sequence (where X is any amino acid except proline). The N-glycosylation process consists of two main steps: (i) synthesis and attachment of the oligosaccharide in the ER (cytosol and endoplasmic reticulum, (ER)), and (ii) maturation by trimming and elongation in both the ER and Golgi [8]. This biosynthesis involves about a hundred enzymes, resulting in a heterogeneous N-glycosylation producing a diversity in sugar structures (glycans) attached to proteins, creating different glycoforms that affect protein function, folding, stability, and immune response. Glycosylation is essential for IgG antibody functionality, as it modulates interactions with immune cells and shapes downstream immune responses. Specific glycan patterns on the Fc region critically influence effector mechanisms such as antibody-dependent cellular cytotoxicity (ADCC), complement-dependent cytotoxicity (CDC), and immune-cell activation [7,9,10,11].

In healthy individuals, more than 95% of IgG molecules are core-fucosylated. The absence of core fucose markedly increases IgG affinity for FcγRIIIa and FcγRIIIb receptors [12,13], resulting in a substantial enhancement of ADCC and phagocytic activity [14,15,16]. Galactosylation also plays a key role: galactose-containing IgG glycans show higher affinity for C1q, thereby promoting classical complement pathway activation [17,18,19]. In addition, galactosylated IgG is specifically recognized by Galectin-3 [20], whereas agalactosylated IgG is enriched in inflammatory and autoimmune conditions [21].

Sialylation further contributes to IgG immunomodulation by enhancing anti-inflammatory activity [22,23]. This effect is mediated through increased binding to the C-type lectin DC-SIGN, expressed on subsets of monocytes, regulatory macrophages, and dendritic cells [24,25]. Importantly, sialylation acts as a molecular switch between pro- and anti-inflammatory IgG functions, particularly in pathogenic autoantibodies [26]. Increasing IgG sialylation reduces antibody pathogenicity and ameliorates disease activity in experimental models, both in vitro and in vivo [26,27,28].

In this study, we describe the IgG N-glycosylation profile of six patients with confirmed RC14 and compare these findings with those from healthy individuals. Notable differences were observed, which may be associated with the increased susceptibility to recurrent infections in these patients, potentially revealing underlying mechanisms and suggesting new therapeutic opportunities.

## 2. Materials and Methods

### 2.1. Patients

This observational cross-sectional study included six patients with RC14 (Table 1). Inclusion criteria: (1) a confirmed diagnosis of RC14 syndrome established through standard cytogenetic analysis and molecular characterization; (2) documented clinical features consistent with the syndrome, including epilepsy, developmental delay/intellectual disability, and ophthalmological abnormalities; (3) availability of adequate serum volume to permit IgG purification and subsequent N-glycan profiling; and (4) informed consent obtained from patients, allowing the use of biological samples for research purposes.

This study was based solely on information and investigations that were carried out as part of the routine clinical care of RC14 patients. All procedures performed in studies involving human participants were in accordance with the ethical standards of the institutional research committee at Policlinico “G. Rodolico-San Marco”, Catania, and with the 1964 Helsinki declaration and its later amendments.

### 2.2. IgG Purification from Whole Serum

IgG was isolated from serum by affinity chromatography using a 96-well Protein G Spin Plate for IgG Screening (c). Prior to use, the spin plate and reagents were equilibrated to room temperature for approximately 30 min. The plate was then mounted on a Positive Pressure-96 Processor (Waters Corporation, Milford, MA, USA), and the resin beads were conditioned with three washes of 200 μL Binding Buffer (BupH™ Phosphate-buffered Saline Packs PBS, pH 7.2, Thermo Scientific, Rockford, IL, USA) under 5–10 mbar pressure. Serum samples (50 μL) were diluted with 150 μL Binding Buffer to maintain a proper ionic strength and pH for optimal binding, then transferred to the wells assembled on a wash plate. IgG binding was promoted by shaking at 1000 RPM for 60 min at room temperature. Then, the plate was mounted on the Positive Pressure-96 Processor, and the solution in the wells drained away. To avoid non-specific binding proteins, the resin was washed four times with 300 μL PBS, discarding the flow-through after each step. The plate was once again removed from the Positive Pressure-96 Processor, and the bottom was gently tapped dry on lint-free paper.

For elution, the purification plate was assembled onto a 96-well collection plate preloaded with 60 μL Neutralization Buffer (1 M Tris-HCl, pH 9, Thermo Fisher Scientific, Waltham, MA, USA) per well to preserve IgG integrity. Bound IgG was eluted twice with 200 μL of 0.1 M formic acid, pH 2.7, (Formic acid ≥ 97.5% eluent additive, for LC-MS, Fluka™, Honeywell Research Chemicals, Seelze, Germany). The purity of the recovered fractions was verified by matrix-assisted laser desorption/ionization mass spectrometry (MALDI-MS). Eluates were subsequently transferred to Eppendorf tubes (Eppendorf Hamburg Germany), dried under vacuum (Savant SpeedVac™ vacuum concentrator Thermo Scientific, Rockford, IL, USA), and reconstituted in 200 μL PBS 20 mM. A 9 μL aliquot of the reconstituted IgG solution was then used for glycan release.

### 2.3. Enzymatic Release of N-Glycans, Labelling and ESI-MS Analysis

The technique used in this study to analyze the N-glycan profile of serum-extracted IgG has been reported as suitable for studying the glycosylation of IgG and monoclonal antibodies [29,30].

Enzymatic release and fluorescent labelling of N-glycans were performed with a GlycoWorks RapiFluor™-MS N-Glycan kit (Waters Corporation, Milford, MA, USA). All reactions and purification steps were performed in the kit’s 96-well plate (Waters Corporation, Milford, MA, USA) format.

Labelled N-glycans, derivatized at the glycosylamine residue of the terminal chitobiose epitope [31,32], were subjected to chromatographic separation using a Vanquish™ UHPLC system (Thermo Scientific™, model 83L6BL3, Waltham, MA, USA). The UHPLC was coupled with a Vanquish™ Fluorescence Detector (Thermo Scientific™, model VC-D50-A), which was further connected in series to an Orbitrap Exploris™ 120 high-resolution mass spectrometer (Thermo Scientific™ Inc., Bremen, Germany). Chromatographic separation was achieved using a hydrophilic interaction liquid chromatography (HILIC) column (ACQUITY UPLC Glycan BEH Amide, 130 Å, 1.0 mm × 150 mm, 1.7 μm; Waters Corporation, Milford, MA, USA). The column operated at 60 °C with a flow rate of 0.1 mL/min. Mobile phase A consisted of 200 mM ammonium formate, pH 4.4 (Ammonium formate solution for glycan analysis, Waters Corporation, Milford, MA, USA), while mobile phase B was acetonitrile. A gradient from 25% to 46% mobile phase A over 35 min was applied.

ESI-MS analyses were performed under the following conditions: ion transfer tube temperature 275 °C, vaporizer temperature 250 °C, spray voltage 3.30 kV. Spectra were acquired in positive ion mode at a resolution of 120,000 FWHM at 200 *m*/*z*. Each sample was analyzed in triplicate, producing nine chromatograms per patient. No substantial intra-sample variation was observed.

### 2.4. Protein N-Glycosylation Analyses by MALDI-MS

N-glycans preparation from whole serum (10 μL) consisted of glycoprotein denaturation by RapiGest™ SF Surfactant (Waters Corporation, Milford, MA, USA) in 50 mM ammonium bicarbonate buffer, followed by reduction in 5 mM DTT (Dithiothreitol, AppliChem, PanReac AppliChem ITW Reagents, Darmstadt, Germany) at 56 °C for 30 min, alkylation in 15 mM IAA (Iodoacetamide, Sigma-Aldrich, Merck KGaA, Darmstadt, Germany) in the dark at room temperature for 45 min, and peptide-N-glycosydase F (PNGase F) digestion (2 U, Roche, Molecular Biochemicals, Mannheim, Germany) overnight at 37 °C. The released glycans were therefore purified by two chromatographic steps on C18 Sep-Pak cartridges (Waters Corporation, Milford, MA, USA) and by solid-phase extraction (SPE) on HyperSep™ Hypercarb™ cartridges (Thermo Scientific™, Bellefonte, PA, USA) [31,33]. The obtained N-glycans were therefore permethylated in a DMSO/NaOH slurry (Dimethyl sulfoxide, Thermo Fisher Scientific, Waltham, MA, USA; Sodium hydroxide pellets, Alfa Aesar, Thermo Fisher Scientific, Ward Hill, MA, USA) followed by CH_3_I addition (Iodomethane, Thermo Fisher Scientific, Waltham, MA, USA), according to the method originally developed by Ciucanu and Kerek [34] to enhance glycan detection sensitivity upon MALDI-MS. Mass spectra of permethylated N-glycans, dissolved in MeOH at an estimated concentration of about 10 pmol/μL, were performed using CMBT (5-chloro-2-mercaptobenzothiazole, Sigma-Aldrich, Merck KGaA, Darmstadt, Germany, 10 mg/mL in 80:20 MeOH/H2O, *v*/*v*) as the matrix [35]. MALDI TOF and MALDI TOF/TOF analyses were conducted on a 4800 Proteomic Analyzer (AB Sciex, Marlborough, MA, USA), equipped with a Nd:YAG laser operating at a wavelength of 255 nm with a <500 ps pulse and a 200 Hz firing rate. Mass spectra of permethylated N-glycans were acquired in positive polarity and in reflector mode, allowing for the detection of monoisotopic masses with mass accuracy below 75 ppm. Data were processed using DataExplorer™ 4.9 software. Structural assignments were based on molecular weight identification, knowledge of the N-glycan biosynthetic pathway and MS/MS analyses. N-glycan species were identified by bioinformatic tools, such as GlycoMod (http://web.expasy.org/glycomod/, accessed on 16 February 2026) and glycoworkbench v2.1 [36], and by tools provided by the consortium for functional glycomics (http://functionalglycomics.org, accessed on 16 March 2026).

### 2.5. Statistical Analysis

Specificity and relevance of quantitative differentiations were established by ANOVA *p*-values, and results were expressed as mean values ± SD. Statistical significance was set at *p* ≤ 0.05.

## 3. Results and Discussion

### 3.1. IgG N-Glycosylation Profiles in RC14 Patients

**Figure 1:**
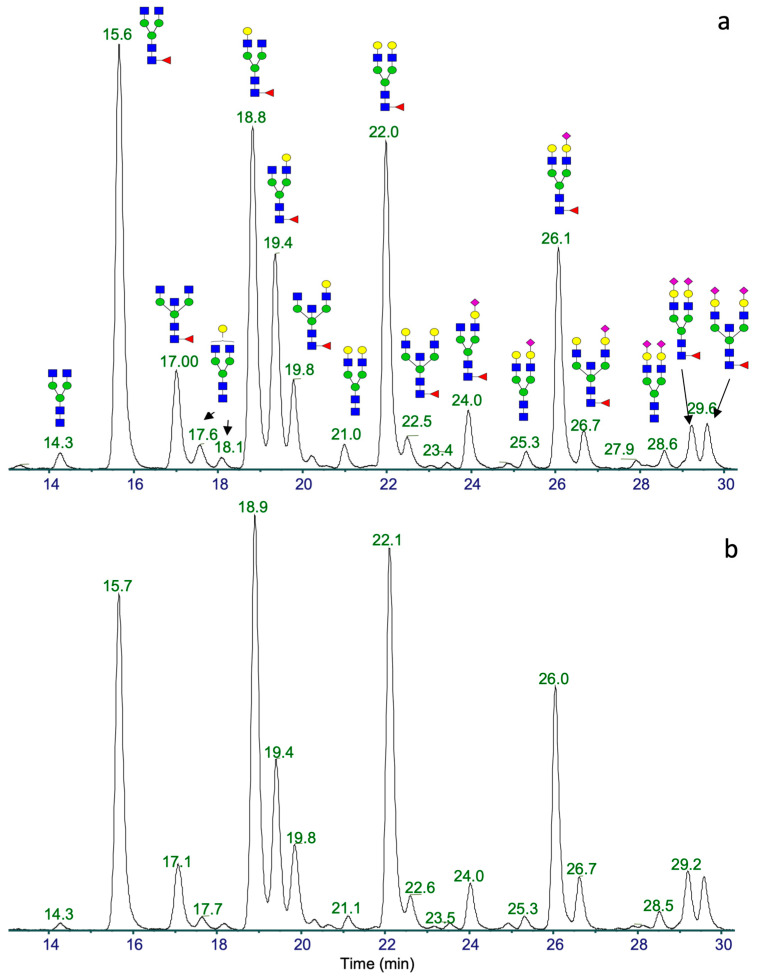
HILIC-UPLC-FLR chromatograms of the IgG N-glycans extracted from patient 1 (**a**) and from a healthy control (**b**). Most abundant N-glycans are detailed and illustrated in (**a**). Glycan symbols: GlcNAc, blue square; Man, green circle; Gal, yellow circle; NeuAc, purple lozenge; Fuc, red triangle.

We used the chromatographic separation of the IgG N-glycans to allow for isomeric structure characterization [29] and high sensitivity, thanks to the fluorescence labelling, with the possibility of unambiguously identifying all the chromatographic peaks by ESI MS and ESI MS/MS. All IgG N-glycans were identified, with the most abundant ones detailed in Figure 1 and in Table 2.

The HILIC-UHPLC-FLR profiles of the IgG N-glycans from RC14 patients show clear differences when compared to age-matched healthy controls, mainly consisting of decreased galactosylation and sialylation (Figure 1a,b and Supplementary Figures S1–S5).

**Figure 2:**
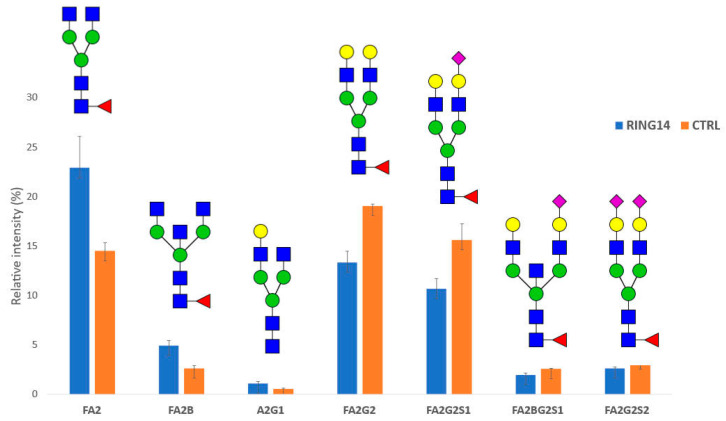
Comparison of statistically significant (*p* < 0.01) relative intensities between control subjects (CTRL) and RC14 syndrome patients. N-Glycans are indicated using the Oxford Notation (see Table 2). Glycan symbols: GlcNAc, blue square; Man, green circle; Gal, yellow circle; NeuAc, purple lozenge; Fuc, red triangle.

The IgG N-glycan profiles among patients are similar but markedly different from controls, showing FA2 (agalactosylated glycan) as the most intense peak, whereas the two most intense species in healthy controls always correspond to FA2G1a (monogalactosylated glycan) and FA2G2 (digalactosylated glycan). Additional differences include increases in bisected and afucosylated N-glycans. ANOVA analysis (Figure 2) reveals seven N-glycans with significant intensity differences: patients exhibit an increase in the afucosylated species A2G1 and the agalactosylated structures FA2 and FA2B, while healthy controls have higher levels of digalactosylated and sialylated glycans. All statistical findings should be interpreted with caution due to the limited number of patients included in the analysis. Despite this constraint, an aberrant IgG glycosylation profile was observed across individuals who present with heterogeneous clinical features. The straightforward explanatory hypothesis may implicate a direct correlation between the aberrant IgG N-glycosylation observed in patients with RC14 syndrome and the underlying chromosomal rearrangement. However, this has yet to be proven, as no genes encoding galactosyltransferases or sialyltransferases are located on chromosome 14. By contrast, the *FUT8* gene, encoding the enzyme α-(1,6)-fucosyltransferase (FUT8) essential for the core fucosylation of N-glycans, maps on chromosome 14q23.3, is usually not involved in rearrangement/deletions leading to typical chromosome 14 ring structures [37]. It is worth noting that haploinsufficiency of the genes included in the deletion is only one of the possible pathogenic mechanisms, which involves several other factors, such as ring chromosome instability during cell division and the effects of the chromosome’s new conformation on gene expression. Therefore, the lack of candidate genes within the region that is typically deleted does not in itself exclude an underlying genetic basis. This is also an element to take into consideration when investigating phenotypic differences among patients.

To investigate a possible generalized N-glycosylation defect owing to a chromosomal defect underlying RC14, we performed the N-glycosylation analysis on total serum proteins and on serum transferrin, one of the most abundant serum liver-derived glycoproteins, used as a valuable biomarker in the diagnosis of Congenital Disorders of Glycosylation (CDG) [38].

**Figure 3:**
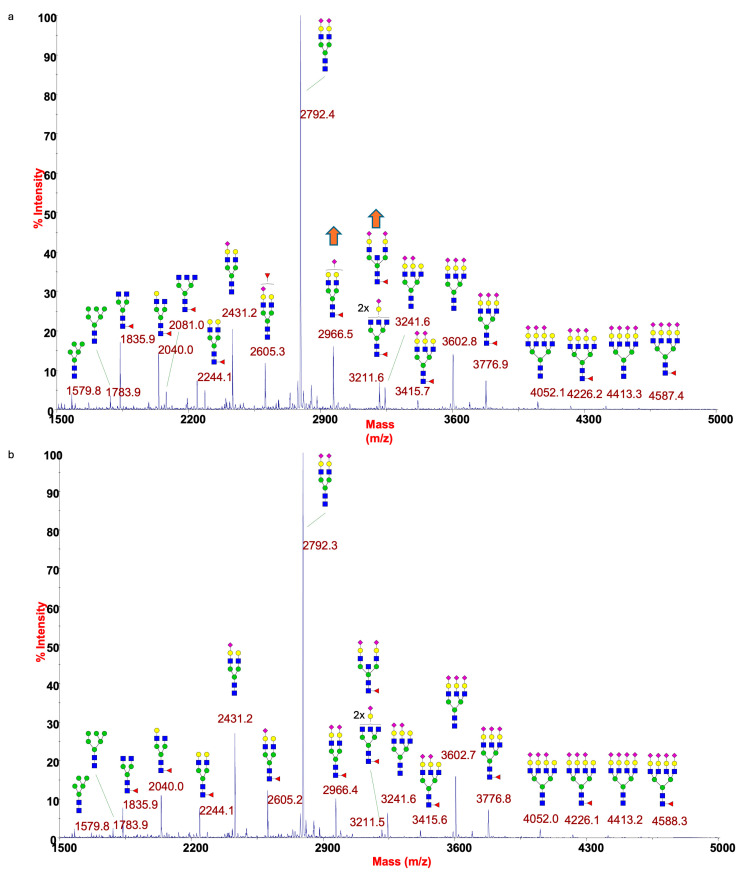
MALDI TOF mass spectrum of serum N-glycans from patient 3 (**a**) and from a healthy subject (**b**). Red arrows indicate increased amounts. Glycan symbols: GlcNAc, blue square; Man, green circle; Gal, yellow circle; NeuAc, purple lozenge; Fuc, red triangle.

The MALDI mass spectra of permethylated N-glycans released from the total serum of patient 3 and of a control subject are shown in Figure 3a and Figure 3b, respectively. No galactosylation or sialylation defects are evident, although some peaks in the 2800–5000 mass range, corresponding to antennary fucosylated N-glycans, are slightly more intense in the RC14 patient when compared to the control. A similar profile was observed for serum and serum transferrin of all the patients included in this study. Since no decreases in galactosylation and sialylation are observed in total serum and serum transferrin N-glycans of patients affected by RC14 syndrome, it appears that the observed increase in agalactosylated and asialylated N-glycans and the concurrent decrease in galactosylated and sialylated glycoforms of serum IgG do not relate to the structural chromosomal RC14 rearrangement. In conclusion, according to present data, the simultaneous increase in agalactosylated and asialylated N-glycans with the concurrent decrease in galactosylated and sialylated glycoforms might enhance the pro-inflammatory IgG structures while decreasing the anti-inflammatory ones. Based on the abnormal IgG glycosylation, patients might be prone to increased inflammatory response, which could explain their recurrent infections as a common feature of RC14 syndrome [2,5], despite serum IgG levels not falling below the normal levels [6].

### 3.2. Comparison with Other Diseases

The observed IgG glycosylation pattern resembles those occurring in autoimmune diseases characterized by a marked increase in undergalactosylated and undersialylated IgG glycoforms [17,18,39,40,41,42].

Rheumatoid arthritis (RA) is one of the most common immune-mediated inflammatory diseases. Over the past 25 years, the management of this condition has been revolutionized, resulting in substantially higher levels of disease remission and better long-term outcomes. RA therapies focus on controlling inflammation and immune response with drugs like conventional DMARDs (methotrexate), IVIG administration [25,27,43,44,45], monoclonal antibodies [46] (adalimumab, etanercept, and tocilizumab), and JAK inhibitors (tofacitinib) [46].

All the above therapies also result in a counterbalancing of IgG glycosylation, restoring the anti-inflammatory glycan pattern [39,47,48,49].

### 3.3. A Preliminary Therapeutic Insight for RC14 Patients?

This evidence would suggest the possibility of therapies with IVIG or monoclonal antibodies to manage untreatable infections in RC14 patients. Among the patients involved in this study, patient 1 received five doses of IVIG (0.7 g/Kg) during a hospital stay for pneumonia. The therapy, along with others, not only proved useful in treating pneumonia, but patient 1 subsequently showed a reduction in seizures from 5–6/week to one/week. Furthermore, regarding RC14, the Understanding Rare Chromosome and Gene Disorders (UNIQUE) website reports that “The research group that recently identified the loss of IGH (Immunoglobulin Heavy Locus) genes as a possible cause of repeated infection also suspects that giving intravenous immunoglobulin may help with seizure control. In this context, the family of a 12-year-old reported to UNIQUE that better seizure control and fewer respiratory infections went hand in hand” [www.rarechromo.org/media/information/Chromosome%2014/Ring%2014%20FTNW.pdf] (accessed on 30 March 2026).

Although IVIG has been occasionally employed in cases of drug-resistant epilepsy [50,51,52] and is considered among the therapeutic options for autoimmune epilepsy [53,54,55,56], these observations should be interpreted with caution. Overall, the findings in RC14 patients receiving IVIG raise the possibility that seizure reduction could, at least in part, be related to modulation of neuro-inflammatory processes associated with altered IgG N-glycan profiles.

In summary, we hypothesize that patients with RC14 syndrome—or at least a subset of them—may exhibit a pro-inflammatory state that predisposes them to severe and prolonged infections, as well as to inflammation-associated neurological manifestations such as epilepsy. If this hypothesis is correct, treatments such as intravenous immunoglobulin (IVIG) and/or monoclonal antibodies could potentially help modulate the inflammatory response, thereby reducing seizure frequency and shortening the duration of infections. However, it is important to emphasize that this remains a theoretical assumption, and these observations should not be interpreted as evidence of therapeutic efficacy.

## 4. Conclusions

The IgG N-glycan profile of patients with RC14 syndrome differs markedly from that of controls, characterized by reduced levels of fucosylation, galactosylation, and sialylation. Notably, one patient showed clinical improvement following IVIG therapy, lending preliminary support to the hypothesis that modulation of IgG glycosylation may represent a potential therapeutic strategy. However, this observation should be interpreted with caution, as the clinical benefit cannot be unequivocally attributed to IVIG treatment alone.

## Figures and Tables

**Table 1:** Summarized clinical data of the patients in this study (Further clinical data can be requested from the corresponding author).

Patient N°	1	2	3	4	5	6
Code	RING11510M	RING13910M	RING38010F	RING13310F	RING23711M	RING12110M
Age (years) at study time	20	30	10	22	14	16
14q deletion size	2 Mb	1.5 Mb	4.95 Mb	0.5 Mb	0.35 Mb	2.5 Mb
Monosomic cells on Peripheral blood	20%	20%	Not available	18%	12%	19%
Age at seizure onset	15 months	2 months	8 months	9 months	3 months	4 months
Epileptic phenotype	Generalized clonic	Generalized tonic–clonic	Generalized clonic	Focal to bilateral tonic–clonic seizures	Generalized tonic–clonic	Clonic
Main infectious events recorded	Pneumonia, sepsis, otitis, sinusitis	Bronchiolitis, otitis, recurrent upper respiratory infections	Bronchiolitis, pneumonia	Lyme disease, otitis, pneumonia, gastroenteritis	Bronchiolitis, urinary tract infections	Otitis, bronchiolitis, pneumonia
Autoimmune disorders	Celiac disease	None	Type 1 diabetes	None	None	None

**Table 2:** Structural and mass information of the IgG N-glycans investigated.

Glycan (Nomenclature)	Structure	[M+2H]^+2^ Calc.	[M+2H]^+2^ Meas.	Error (ppm)
A2	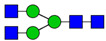	814.8378	814.8380	0.25
FA2	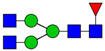	887.8668	887.8679	1.24
FA2B	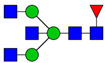	989.4065	989.4071	0.61
A2G1a	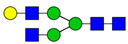	895.8643	895.8649	0.67
A2G1b	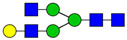	895.8643	895.8649	0.67
FA2G1a	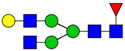	968.8932	968.8932	0.00
FA2G1b	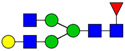	968.8932	968.8945	1.34
FA2BG1a	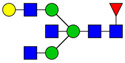	1070.4329	1070.4334	0.47
A2G2	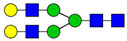	976.8907	976.8918	1.13
FA2G2	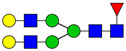	1049.9196	1049.9207	1.05
FA2BG2	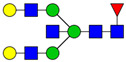	1151.4593	1151.4619	2.26
FA2G1S1	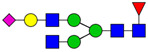	1114.4409	1114.4427	1.62
A2G2S1	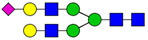	1122.4384	1122.4396	1.06
FA2G2S1	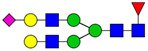	1195.4673	1195.4698	2.09
FA2BG2S1	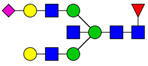	1297.0070	1297.0097	2.08
A2G2S2	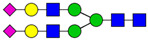	1267.9861	1267.9880	1.49
FA2G2S2	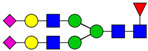	1341.0150	1341.0183	2.46
FA2BG2S2	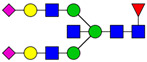	1442.5547	1442.5574	1.87

## Data Availability

Raw files were uploaded to Glycopost (GPST000705) (https://glycopost.glycosmos.org/, accessed on 16 March 2026).

## Supplementary Materials

The following supporting information can be downloaded at: https://www.mdpi.com/article/10.3390/biom16060760/s1, Figures S1–S5 “HILIC-UPLC-FLR chromatograms of the IgG N-glycans extracted from patients 2–6”. Figure S1: HILIC-UPLC-FLR chromatograms of the IgG N-glycans extracted from patient 2; Figure S2: HILIC-UPLC-FLR chromatograms of the IgG N-glycans extracted from patient 3; Figure S3: HILIC-UPLC-FLR chromatograms of the IgG N-glycans extracted from patient 4; Figure S4: HILIC-UPLC-FLR chromatograms of the IgG N-glycans extracted from patient 5; Figure S5: HILIC-UPLC-FLR chromatograms of the IgG N-glycans extracted from patient 6.
