# Supplementary material for: IgG Glycosylation Analysis in Patients with Ring14 Syndrome Unveils Novel Pathomechanisms and New Therapy Perspectives
Source: Biomolecules. 2026 May 22;16(6):760. doi: 10.3390/biom16060760 (PMC13297435; doi:10.3390/biom16060760)
Supplement: Supplementary file 1 [file biomolecules-16-00760-s001.zip › biomolecules-4263897-supplementary.pdf]

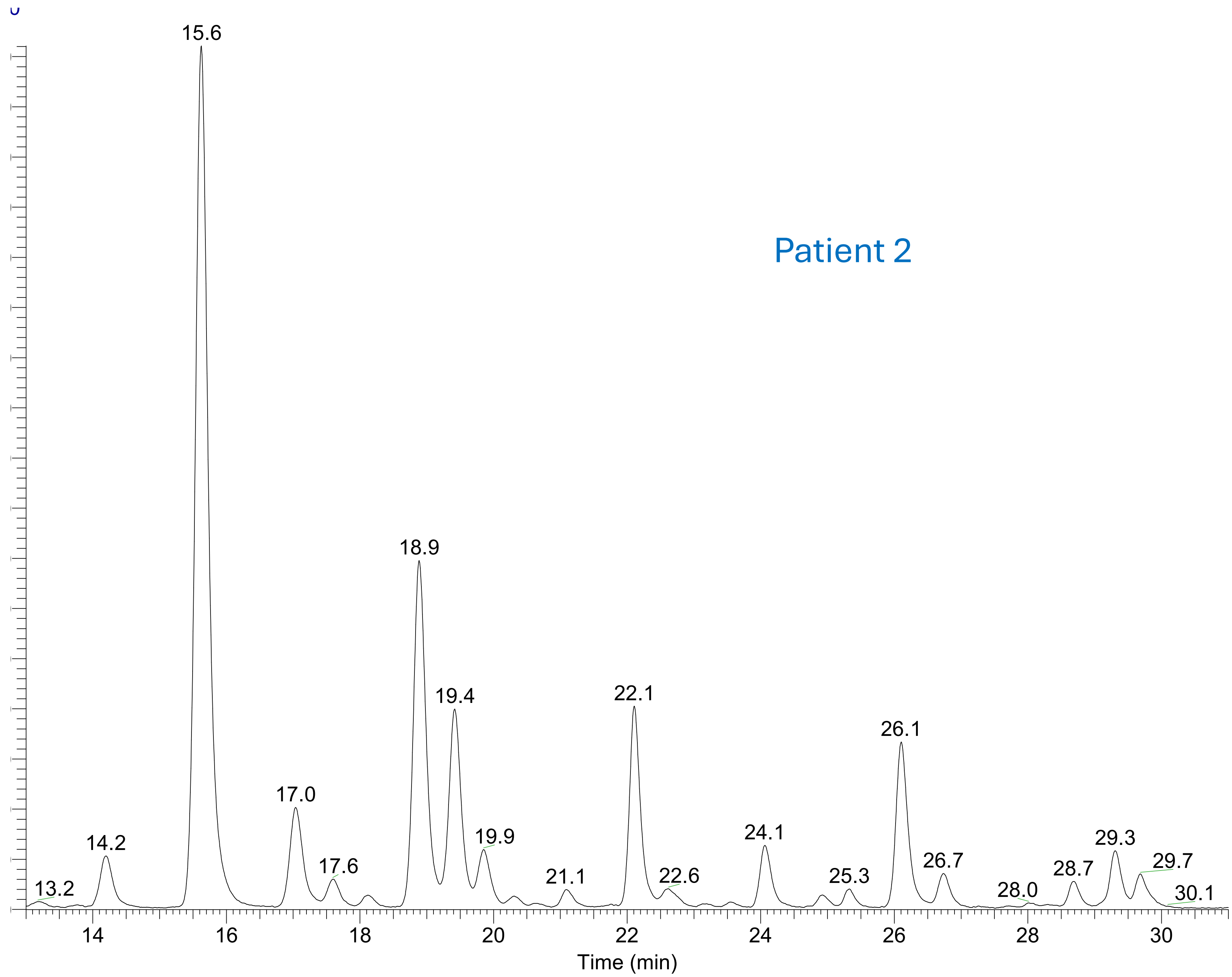

Figure S1. HILIC-UPLC-FLR chromatograms of the IgG N-glycans extracted from patient 2

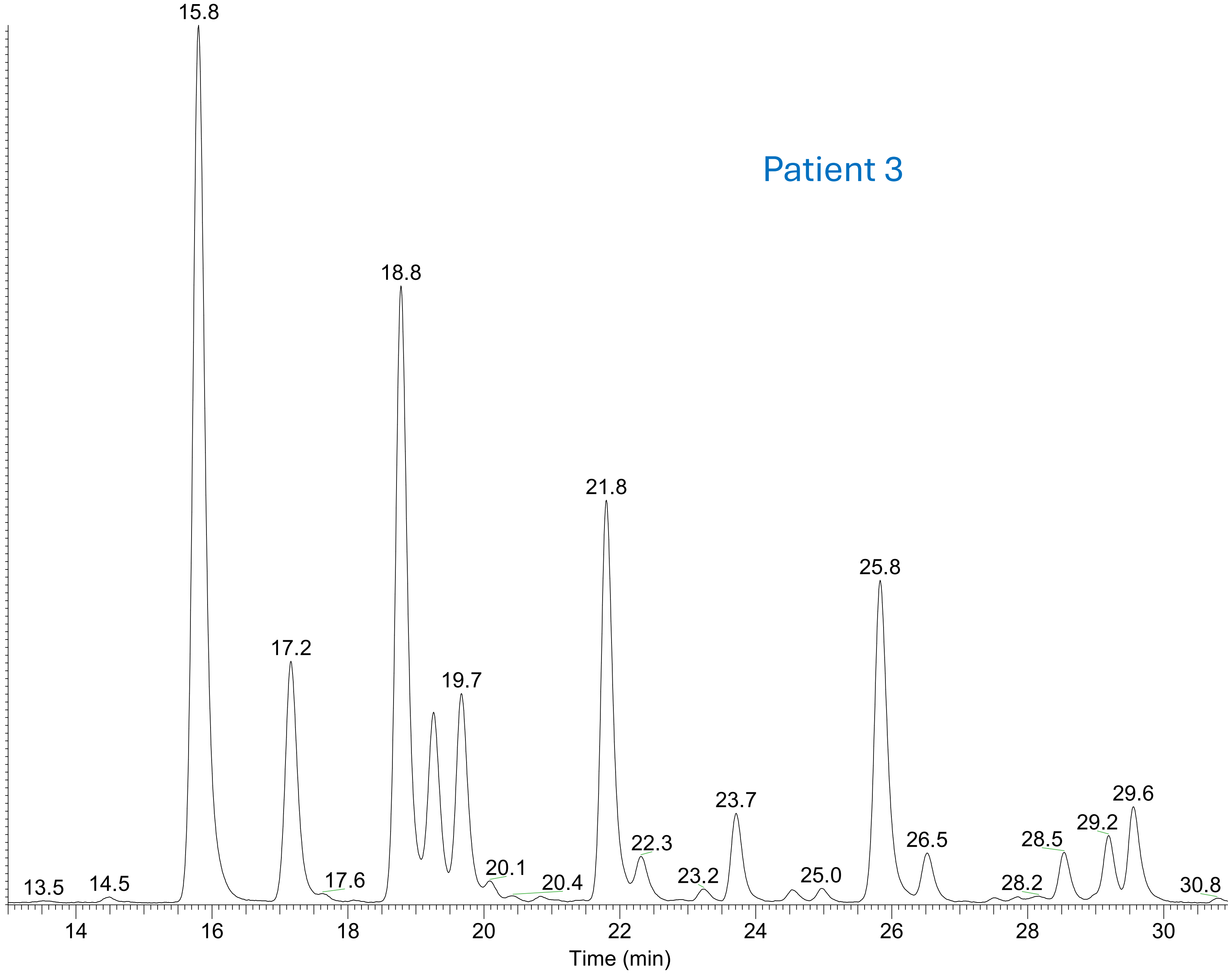

Figure S2. HILIC-UPLC-FLR chromatograms of the IgG N-glycans extracted from patient 3

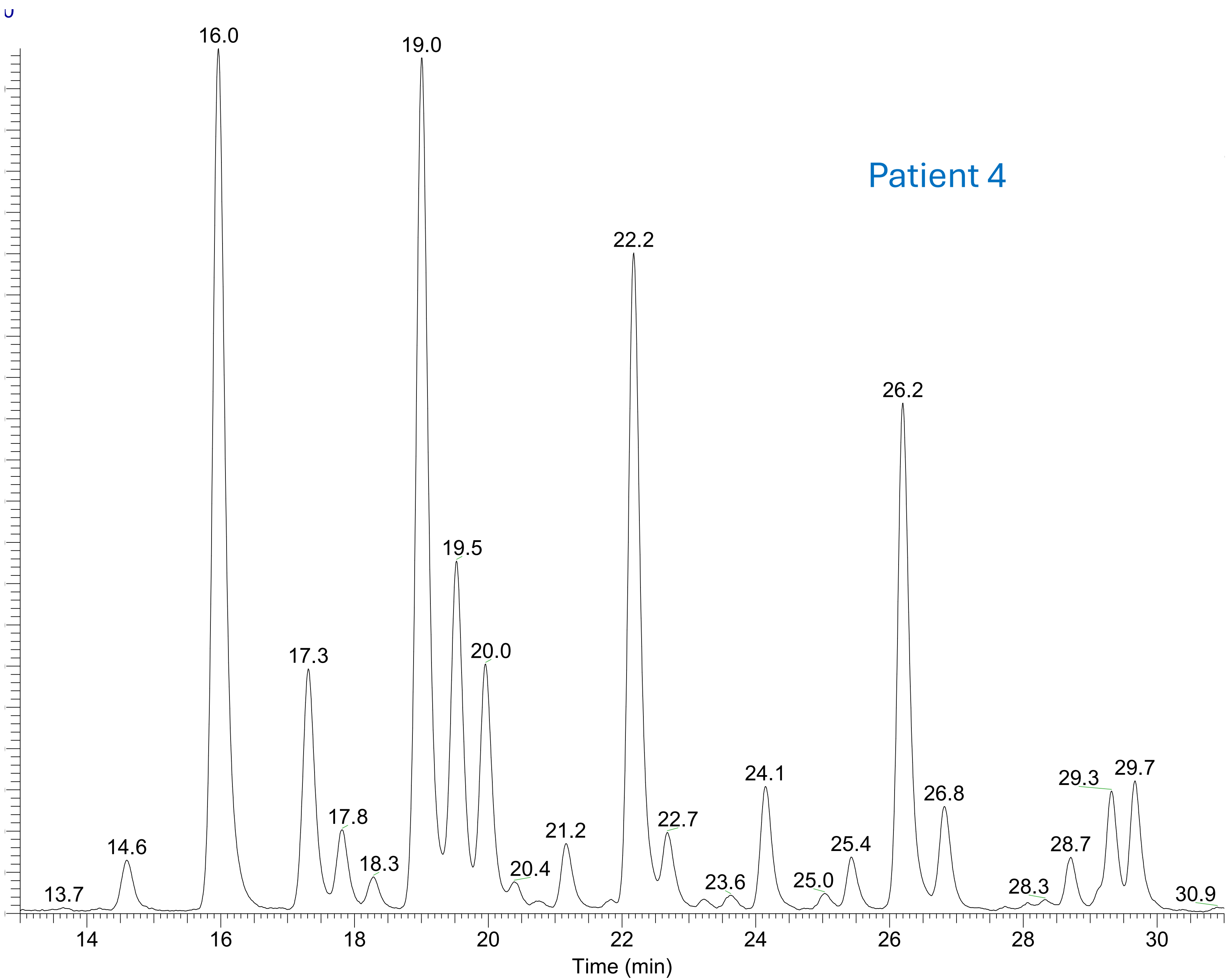

Figure S3. HILIC-UPLC-FLR chromatograms of the IgG N-glycans extracted from patient 4

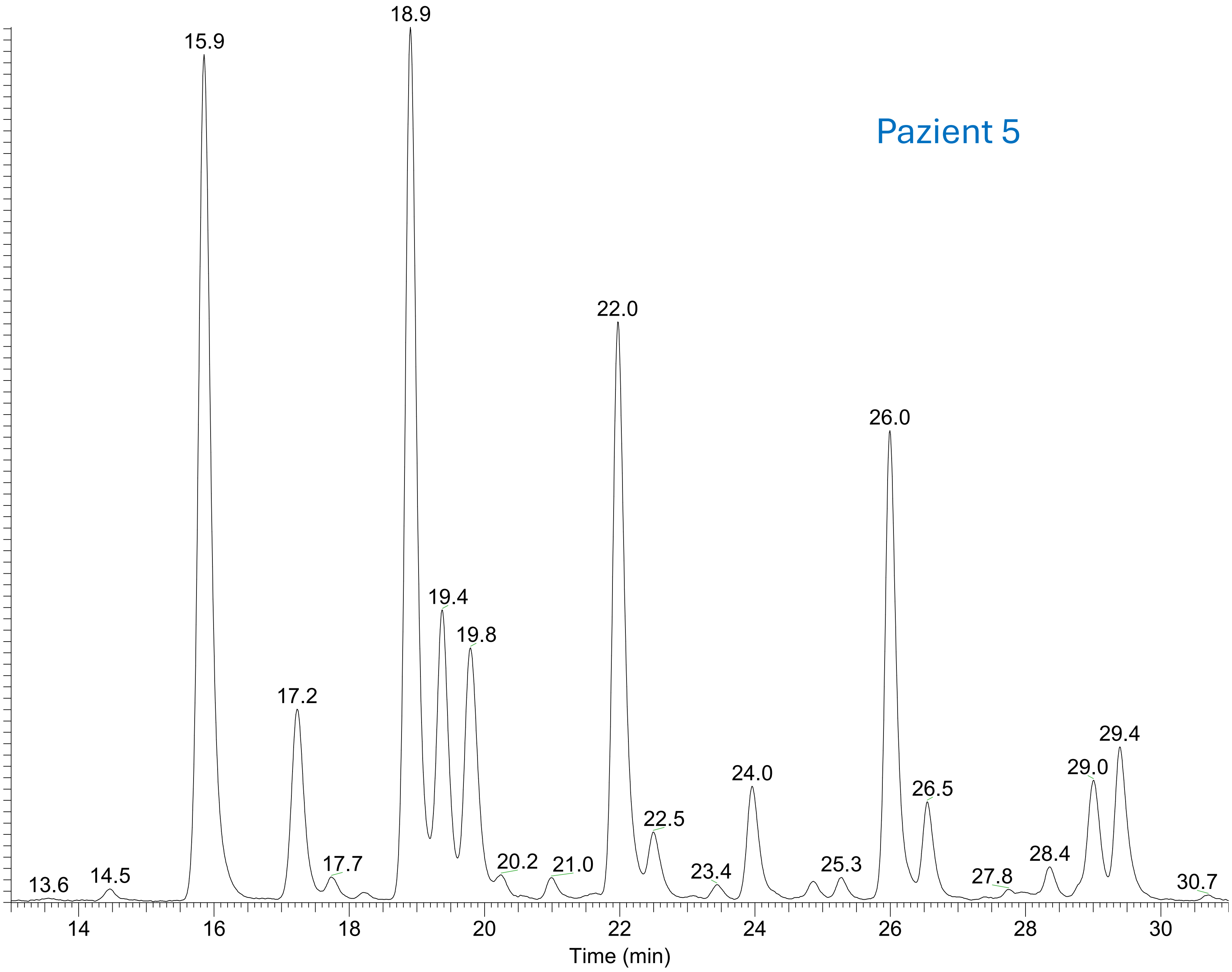

Figure S4. HILIC-UPLC-FLR chromatograms of the IgG N-glycans extracted from patient 5

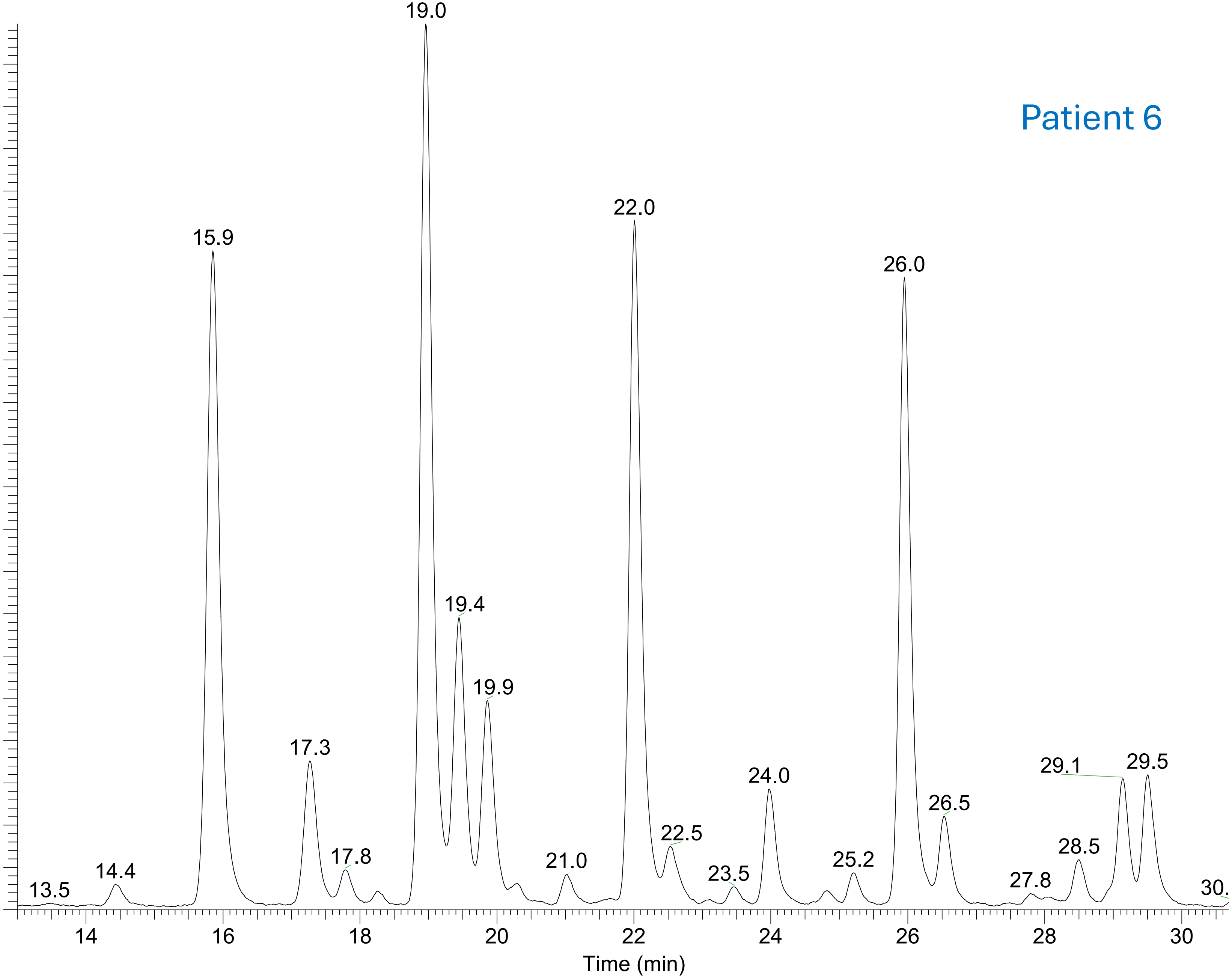

Figure S5. HILIC-UPLC-FLR chromatograms of the IgG N-glycans extracted from patient 6
